# Hydropathy

**Published:** 1842-11-26

**Authors:** 


*Hydropathy.*—M. Scotoutten, of Strasbourg, has received a commission from the French government to visit the hydropathic establishments of Germany, and report on the real merits of the modes of treatment therein practised.—*Prov. Med. Jour.*, Oct. 29, 1842.

